# Biosensors Used for Epifluorescence and Confocal Laser Scanning Microscopies to Study *Dickeya* and *Pectobacterium* Virulence and Biocontrol

**DOI:** 10.3390/microorganisms9020295

**Published:** 2021-02-01

**Authors:** Yvann Bourigault, Andrea Chane, Corinne Barbey, Sylwia Jafra, Robert Czajkowski, Xavier Latour

**Affiliations:** 1 Laboratory of Microbiology Signals and Microenvironment (LMSM EA 4312), University of Rouen Normandy, 55 rue Saint-Germain, F-27000 Evreux, France; yvann.bourigault@univ-rouen.fr (Y.B.); chane.andrea@gmail.com (A.C.); corinne.barbey@univ-rouen.fr (C.B.); 2 Research Federations NORVEGE Fed4277 & NORSEVE, Normandy University, F-76821 Mont-Saint-Aignan, France; 3 Division of Biological Plant Protection, Intercollegiate Faculty of Biotechnology UG and MUG, University of Gdansk, ul. A. Abrahama 58, 80-307 Gdansk, Poland; sylwia.jafra@biotech.ug.edu.pl; 4 Division of Biologically Active Compounds, Intercollegiate Faculty of Biotechnology UG and MUG, University of Gdansk, ul. A. Abrahama 58, 80-307 Gdansk, Poland

**Keywords:** fluorescent proteins, green fluorescent protein (GFP), microscopy, plant colonization, *Dickeya*, *Pectobacterium*, *Erwinia*, potato blackleg and soft-rot, biocontrol

## Abstract

Promoter-probe vectors carrying fluorescent protein-reporter genes are powerful tools used to study microbial ecology, epidemiology, and etiology. In addition, they provide direct visual evidence of molecular interactions related to cell physiology and metabolism. Knowledge and advances carried out thanks to the construction of soft-rot *Pectobacteriaceae* biosensors, often inoculated in potato *Solanum tuberosum*, are discussed in this review. Under epifluorescence and confocal laser scanning microscopies, *Dickeya* and *Pectobacterium*-tagged strains managed to monitor in situ bacterial viability, microcolony and biofilm formation, and colonization of infected plant organs, as well as disease symptoms, such as cell-wall lysis and their suppression by biocontrol antagonists. The use of dual-colored reporters encoding the first fluorophore expressed from a constitutive promoter as a cell tag, while a second was used as a regulator-based reporter system, was also used to simultaneously visualize bacterial spread and activity. This revealed the chronology of events leading to tuber maceration and quorum-sensing communication, in addition to the disruption of the latter by biocontrol agents. The promising potential of these fluorescent biosensors should make it possible to apprehend other activities, such as subcellular localization of key proteins involved in bacterial virulence *in planta*, in the near future.

## 1. Introduction

Soft-rot *Pectobacteriaceae* (SRP) [1] consist of the bacterial genera *Pectobacterium* and *Dickeya*, whose members are responsible for a wide spectrum of soft-rot diseases of agriculturally important vegetable crops and ornamental plants worldwide [2,3,4]. Currently, the *Pectobacterium* genus encompasses nearly twenty species, while the *Dickeya* genus contains ten species [5,6,7,8,9,10,11,12,13]. The ecological niches of SRP bacteria are various; they can be isolated from plant, bulk and/or rhizosphere soil [14], surface and ground water, such as the most recently defined novel species *Dickeya lacustris* and *Dickeya undicola* [6,15,16], and on the surface and interior of insects [17,18,19]. Differentiated by their genetic content and virulence potential, most SRP species provoke significant losses in crop production of potato, carrot, tomato, pineapple, maize, rice, hyacinth, and chrysanthemum *inter alia* [20]. In potato (*Solanum tuberosum* L.), the fourth main food crop worldwide [21], these bacteria are responsible for blackleg of field-grown plants and tuber soft-rot in the field, storage, and transit. As such, *Pectobacterium* spp. and *Dickeya* spp. are considered to be among the top ten most important agricultural bacterial phytopathogens worldwide [22].

Despite the seedborne nature of SRP diseases in potato, these bacteria are facultative saprophytes, present in the soil and ground water that can switch to pathogenic behavior when they encounter susceptible plant hosts [4,10,14]. SRP bacteria may invade surrounding plants via wound sites or natural openings, such as lenticels or stomata, causing latent infection without developing disease symptoms [4]. During this hidden phase of the infection cycle, bacteria can survive in a dormant state for a long time inside vascular or parenchymatous tissues, representing the most important source of disease inoculum [23,24,25,26], but not the sole mode of transmission according to spatial analysis of blackleg-affected seed potato crops in Scotland [27]. Vascular vessels lead to the propagation of pathogens throughout the whole plant and progeny tubers. In the vascular vessels, *Pectobacterium atrosepticum* and *Pectobacterium brasiliense* (formerly *P. carotovorum* subsp. *brasiliense*) form specific multicellular structures, named “bacterial emboli”, causing vessel occlusion of potato and tobacco plants for downward migration of the bacteria [28,29]. The formation of these biofilm-like aggregates, the matrix of which is composed of plant pectic polysaccharides, seems to be a universal strategy of *Pectobacterium* spp. interactions with host plants [28]. When certain environmental conditions (high humidity, fresh or mild temperatures, and poor oxygen availability) become favorable for plant disease, *Dickeya* and *Pectobacterium* species undergo a transition from latent to pathogenic [14]. This pathogenic phase is characterized by rapid bacterial multiplication and the production of numerous extracellular lytic enzymes, involved in invading and destroying plant tissues, responsible for the necrotrophic lifestyle of these pathogens [30,31]. The production of these virulence factors is regulated by a LuxI/LuxR type quorum-sensing (QS) system for both *Pectobacterium* and *Dickeya* genera [32,33,34,35,36,37], and the virulence factor modulating (Vfm) system found in the genus *Dickeya* [38] ensures successful infection by preventing premature activation of plant defenses.

The transmission of disease, chronology of disease progression, and pathogenicity mechanisms have been well studied in SRP [23,24,25,30,39]. However, knowledge is limited by the paucity of direct visual evidence of these mechanisms *in planta*. To beat these shortcomings, reporter strains expressing fluorescent markers, selected among a wide range of fluorescent proteins (FPs), were developed to study both survival and viability of SRP in sit*u*, as well as to monitor systemic colonization of host plants during infection. An additional degree of complexity is provided when the virulent activity of SRP is additionally monitored by secondary FPs and/or when biocontrol agents, also carriers of FPs, are co-inoculated with SRP into the plant. This not only makes it possible to follow the localization of each partner but also their pathogenic or protective activities carried out with respect to plant development and fitness.

## 2. Main Traits of Fluorescent Proteins Used to Construct Tagged-Soft-Rot *Pectobacteriaceae*

### 2.1. Fluorescent Proteins: Origin, Structure, Spectral Range, and Stability

In the last 50 years, FPs have become a very useful tool to monitor bacterial activities by giving them a specific phenotype that is easily detectable in situ. The majority of FPs originate from *Hydrozoa* and *Anthozoa* species, which are recognized for their ability to emit various biological luminescence [40]. The most well characterized FP is the green fluorescent protein (GFP). It was discovered and isolated from the jellyfish *Aequorea victoria* by Osamo Shimomura and co-workers in 1961, themselves rewarded for this in 2008 by the Nobel prize [41,42]. The first use of the *gfp* gene as a marker of gene expression in *Escherichia coli* dates back to 1994 [43]. Then, a wide range of eukaryotic or prokaryotic organisms, including mammals, fish, insects, plants, and of course, microorganisms, such as yeasts and bacteria, were tagged with GFP [44]. In addition to gene expression studies, GFPs allow promoter tracking, communication detection, protein labeling, subcellular localizations, photobleaching techniques, nucleic acids, and cell and tissue labeling [40,45,46].

GFPs are polypeptides with a molecular weight of about 25 kDa. They are composed of a single domain protein of 220–240 amino acids [40,47]. Three key amino acid residues, located between positions 65 to 67, are responsible for the chromophore formation consisting of a 4-(4-hydroxyphenyl) methylideneimidazole-5-one group [42,48]. The fluorescent mechanism results from a two-step reaction: first, an autocatalytic cyclization of the chromophore part, followed by dehydrogenation in the presence of oxygen [40,47]. Therefore, for green light emission, GFP needs oxygen but does not require complex formation or polymerization, nor ATP consumption (or other cofactors), limiting interference with other cellular and molecular processes in the host [44,49]. Two major absorption bands are characteristic for the maturated GFP protein: a major band at 395 nm and a minor band at 475 nm. Based on these two excitation wavelengths, one major emission wavelength is observed at 508 nm [40,42].

Several GFP-like proteins exist and emit a wide range of color radiations in addition to green. A list of these FPs diversity that are currently available for cell labeling is proposed in the Supplementary Materials (Table S1 [50,51,52,53,54,55,56,57,58,59,60,61,62,63,64,65,66,67,68,69,70,71,72,73,74]). Briefly, five main spectral classes of FPs can be considered: cyan (excitation ≈ 450 nm, emission ≈ 485 nm), green (excitation between 480 and 510 nm, emission between 500 and 520 nm), red (excitation between 560 and 580 nm, emission between 570 and 610 nm), green to red photoconvertible (same spectral region but emission between 620 and 630 nm), and purple-blue nonfluorescent protein (single absorption peak between 560 and 610 nm). This latest FP class, which is by definition nonfluorescent, will produce bright fluorescence when exposed to intense green light irradiation [75,76]. This variety of FPs is the consequence of the diversity of chromophore structures with several origins [77]. Some chromophore variants could appear “naturally”, while others could be engineered in labs by amino acid residue mutation or substitution [40]. To date, according to Remington [48], there are seven naturally occurring chromophores: GFP, DsRed, mTagBFP, zFP538, mO or mKO, KFP, and Kaede. These chromophores derivate from a single protein that undergoes different modifications to give these specific chromophores. A single substitution of the Thr residue at position 203 by Tyr in the wild-type GFP sequence creates the yellow fluorescent protein (YFP) [48]. For blue fluorescent protein (BFP) and cyan fluorescent protein (CFP), the Tyr residue in GFP was replaced by His and Trp residues, respectively [78]. In some cases, mutations are used to reduce photobleaching or signal interference. For example, modifications of GFP to obtain the red shifted GFP with an excitation wavelength of 481–501 nm lead to a 20–30-fold fluorescence signal increase and a reduction of photobleaching at 395 nm [49].

Obviously, the use of FPs has limits and requires some improvements. A major drawback of FPs is their stability. Once produced, the GFP is extremely stable and could persist a long time in the host, rending difficult the studies of real-time gene expression, for example. Thus, the need to develop new variants, which are less stable, appears necessary [44]. The GFP C-terminal region was defined as the best part for the construction of new variants with high susceptibility toward the action of proteases, causing a decrease in their stability [44]. These variants are obtained by adding a peptide tag encoding DNA sequence at the 3′ end of the *gfp* gene. This peptide tag is recognized by housekeeping or intracellular tail-specific proteases, allowing quick degradation of the modified GFP [45]. A series of unstable variants of GFP with different half-lives ranging from 40 min to a few hours were constructed by slightly modifying the DNA sequence encoding the C-terminal peptide tag, such as GFP(LAA), GFP(AAV), and GFP(LVA) [44].

Several factors could affect FP sensitivity. Firstly, the chromosomal or plasmid location of the FP encoding gene may greatly affect fluorescence intensity because of the presence of several copies of this gene per cell, according to the plasmid copy number, contrary to the single chromosomal copy. Furthermore, the choice of promoter for tuning the FP encoding gene expression, is also crucial. Indeed, the promoter strength depends, *inter alia*, on the sequence of the RNA polymerase binding sites and may be considered for modulating gene expression to obtain good fluorescence intensity. According to the intended applications, FP encoding genes may be constantly expressed, regardless of the experimental conditions, by using a constitutive promoter. Conversely, the use of an inducible promoter leads to the transient expression of these genes in a specified time period. The level of FP production exhibited by a bacterial cell depends on the strength of ribosomal binding sites and degradation rates of the protein products. Due to the risks of plasmid transfer to other organisms, a chromosomal tag could ensure genetic stability. However, a single copy of GFP is not enough to allow the effective detection of fluorescence. To counteract this problem, Tn*5* or Tn*10*-based transposon suicide delivery vectors can be used to mark various bacteria [49]. Solutions to allow better detection of fluorescence are the use of strong promoters or mutants with enhanced fluorescence intensity.

### 2.2. Interests of Fluorescent Proteins for Studies of Plant-Bacteria Interactions

Several rhizobacteria have been tagged by FPs to study their fitness and impacts on roots or toward other members of the rhizosphere [79,80,81,82,83]. In this context, a major interest of FPs in biosensor development is to monitor the location of targeted rhizobacteria and their distribution on and inside the plant. This type of tag remains easy to implement. The main requirement is to introduce the FP encoding gene under the control of a constitutive promoter to continuously produce the FP. Thus, fluorescence is always produced and detection of the rhizobacteria remains possible over long periods. One often-used promoter is *T7*, which is constitutive. Study of the *Rhizobiaceae*-leguminous symbiosis can be cited as an example among the first rhizosphere applications of FPs. Indeed, *Sinorhizobium meliloti* (formerly *Rhizobium meliloti*) strain MB5OI was marked with GFP-S65T under the control of the *Salmonella typhimurium trp* promoter, another constitutive promoter, to study rhizobial fitness and plant infection stages [49,84]. This work allowed the visualization of symbiosis induction, characterized by initial key infection between *S. meliloti* and alfalfa roots. Furthermore, it determined the growth rate of *S. meliloti* inside the host plant, which remained slow or outside the roots, depending on the bacterial location under the base or tip of the root. Fluorescent labeling revealed, in this example, the control of bacterial growth by plants through limiting nutrient availability. Then, the same tag was used on non-symbiont plant-growth promoting rhizobacteria (PGPR), *Pseudomonas fluorescens* WC5365 and A506. Here, FPs allowed the visualization of pseudomonad colonization of tomato and *Lotus japonica* roots among native bacteria, specifically in root areas emitting high exudation [82,85,86]. Furthermore, the use of several rhizosphere stable vectors, expressing FPs with different colors, labeled different bacterial populations in the same ecological niche, thus, making it possible to study bacterial interactions [80]. As an example, the biocontrol agent (BCA), *P. fluorescens* DR54-BN14, was tagged with GFP to assess its colonization and behavior in the barley rhizosphere. In this study, *P. fluorescens* DR54-BN54 colonized the root surface and persisted to an elevated density (1.10^7^ CFU.g^−1^ of dry root after 41 days). Moreover, this BCA formed microcolonies associated with the indigenous bacteria already present, which is a behavior already known in other pseudomonads from gnotobiotic systems [82]. The capacities of these biosensors can be increased by multiple labeling. Thus, *Pseudomonas protegens* (formerly *P. fluorescens*) CHA0, another BCA, was dual-tagged with mCherry and GFP. mCherry is a simple tag of the bacteria used to assess localization and root colonization, whereas the GFP tag allows simultaneous recording of antifungal gene expression [83].

Of course, these fluorescent tools were also successfully applied for studies concerning phytopathogens, such as *P. syringae* [87,88], *Xanthomonas campestris* [89,90,91], *Ralstonia solanacearum* [92,93,94], and *Clavibacter michiganensis* [95,96]. Nevertheless, to our knowledge, it has only been since 2010 that FPs have been used to label SRP, leading to significant progress in the knowledge of their interactions with the host plant (Table 1). These elements are addressed in the following sections.

## 3. Colonization of Plants by Fluorescent Protein-Tagged *Dickeya* spp. and *Pectobacterium* spp.

In the case of SRP, fluorescently labeled *Pectobacterium* and *Dickeya* spp. strains have been in the majority, developed and used to study survival and viability of these pathogens in various environments and specifically to examine their behavior inside infected plants [106]. Likewise, the tagged SRP strains have been employed together with microscopic observations to follow the fate and spread of individual SRP cells, as well as the microcolonies of bacteria inside host plants during infection development [97,98].

To study SRP viability *in planta* and monitor the progress of infection, an individual SRP strain is usually transformed with a high copy plasmid carrying a constitutively expressed gene coding for a fluorescent protein. This strategy allows a consistently high level of expression of the fluorescent protein inside the target *Pectobacterium* and/or *Dickeya* spp. cells under virtually all environmental conditions [98,101]. The main advantage of using a plasmid-encoded fluorescent protein is that the introduction of such a plasmid to the cells does not result in the modification of the bacterial chromosome and, hence, it is unlikely that its presence may affect global gene expression regulation of the target bacterium. Nevertheless, the major drawback of this type of cell tagging is that the plasmid carrying a fluorescent protein gene may be lost when bacteria multiply in the environment without selection. Therefore, a plasmid that is easily maintained in the cells without selection is preferred to be used for such purposes [98,101]. By far, as is also reflected in this review, in the majority of studies concerning the SRP-plant interactions performed so far in fluorescently tagged SRP bacterial cells, GFP has been used.

One of the first examples in which fluorescently-labeled SRP strains were used in plant-microbe interactions studies was the work of Golan et al. [106], in which the authors tagged *P. carotovorum* subsp. *carotovorum* Pcc3 and Pcc13 strains with a GFP to monitor the presence and spread of the tagged bacteria in *Ornithogalum dubium* (sun star or star of Bethlehem) plantlets. This study combined microscopic observations with fluorescent activated cell sorter (FACS) analysis to rapidly quantify bacterial cells *in planta* [106]. The assessment of bacterial cell number with FACS accurately correlated the cell numbers evaluated via selective plating, which indicated that fluorescent protein tagging coupled with FACS analysis can be routinely used to precisely quantify bacterial densities in complex environments.

**Figure 1:**
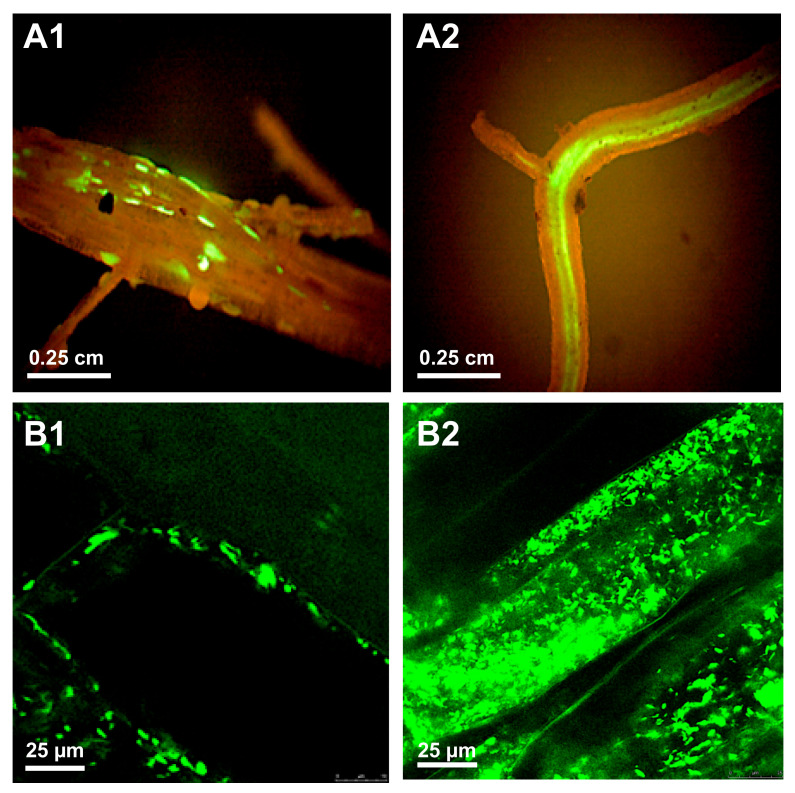
Colonization of potato roots with green fluorescent protein (GFP)-tagged *Dickeya solani.* Bacterial colonization of roots can be evaluated using either epifluorescence stereo microscopy to visualize the spread of the *D. solani strain* IPO2254 (see Table 1) on the scale of the organ (microcolonies on the rhizoplane and invasion of the vascular tissue, (**A1**,**A2**), respectively) or by confocal scanning laser microscopy to locate the bacterium on the level of the plant cell (intercellular and intracellular colonization, (**B1**,**B2**), respectively). For technical experimental details please see reference [97]. (Courtesy of Dr. Jan van der Wolf, WUR-PRI, Wageningen, the Netherlands).

In other studies, Czajkowski et al. [97,98] used a GFP-tagged *Dickeya solani* combined with fluorescent stereomicroscopy and confocal laser scanning microscopy (CLSM) techniques to investigate whether soil infestation or stem/leaf inoculation with the bacteria *D. solani* was able to systematically spread inside potato plants (Figure 1). In those studies, it was shown for the first time that *D. solani* is not only able to use xylem vessels to move upward and downward from the inoculation sites in potato plants, but the bacterium can easily and quickly move to distantly located plant tissues without extensive manifestation of symptoms. In follow-up studies, Fikowicz-Krosko and Czajkowski [107] used a GFP-tagged *D. solani* to assess the bacterial colonization pattern on a bacterial secondary host, weed plant *Solanum dulcamara* (bittersweet nightshade). The obtained results suggested that *D. solani* colonizes potato and *S. dulcamara* plants in a very similar manner, as it was evidenced after infection that the bacteria used vascular tissue to systemically spread inside both plant species and cause visually similar symptoms in culture-tube grown plants [107]. Recently, Kastelein et al. [104] assessed the spread of GFP-tagged *D. solani* and *Pectobacterium parmentieri* strains inside potato plants after spray-inoculation of wounded and intact potato leaves. The GFP-tagged bacteria belonging to both species were detected in distantly located plant parts, indicating that upon favorable conditions, the SRP bacteria can probably pose a risk for the next generation of crop cultivation [104]. In the aforementioned studies, GFP tagging was done to support the observations on the spread of bacterial pathogens in host plants.

One of the examples in which fluorescent proteins other than GFP were used to label the SRP cells is the study done by Kubheka et al. [101], in which mCherry was used to tag *P. brasiliense* cells. This study was conceived to monitor the fate of the bacterium in SRP-susceptible and -tolerant potato cultivars upon their infection with bacteria [101]. In both susceptible and tolerant cultivars, the bacteria were able to colonize the roots; however, the differences were observed in the systemic spread of the bacteria inside plants. In the susceptible cultivar, mCherry-tagged *P. brasiliense* was able to form biofilms and occlusions in xylem vessels in infected tissues, whereas in the tolerant potato cultivar, the tagged bacteria were visualized as free-swimming fluorescent cells present in different tissues of the plant but neither in large numbers nor forming biofilm and occlusion structures [101].

There are also, although limited, examples in which the fluorescently-tagged SRP was used to investigate processes other than colonization of plant tissues and/or bacterial spread inside plants. For example, Czajkowski et al. [108] used GFP-tagged *D. solani* to assess whether treatment of the culture-tube grown potato plants with salicylic acid may protect plants from rotting caused by the bacterium [108]. For this purpose, salicylic acid-treated plants were inoculated with GFP-tagged *D. solani,* and the presence of the bacteria and their spread in roots of inoculated plants was monitored with the use of CLSM. Using this technique, it was possible to evidence the presence of GFP-labeled *D. solani* on the surface of the roots of control plants but not on the surface of the roots of plants treated with salicylic acid.

Similarly, Moleleki et al. [29], when assessing the impact of the *N*-acyl-L-homoserine lactones (AHLs)-driven QS mechanism on the virulence of *P. brasiliense* strain 1692 (Table 1), used GFP-tagged bacterial cells to unravel the phenotype of the AHLs-deficient mutant *in planta* [29]. The authors showed that the GFP-tagged AHLs-deficient mutant was able to form a biofilm inside the intercellular zone of plant tissues, but in a different way to the wild-type strain, and it was unable to occlude the xylem vessels. Furthermore, the mutant was defective in flagella synthesis and swimming motility—phenotypes important for infection of host plants under natural conditions. Likewise, Narváez-Barragán et al. [105] used FPs-tagging of *P. brasiliense* strains (Table 1) to determine the size of bacterial colonies for swarming analyses, which was a part of the phenotypic studies to analyze newly isolated SRP strains, in addition to the cyclic diguanylate (c-di-GMP) intracellular production. C-di-GMP is a bacterial second messenger of growing recognition involved in the regulation of a number of complex physiological processes like the transition from motile to sessile lifestyle [109]. In this study, the cytosolic abundance of cyclic diguanylate (c-di-GMP) was monitored using the plasmid pFY4535 (Table 1). The latter encodes a c-di-GMP bio-sensor consisting of two fluorescent reporters: AmCyan with constitutive expression, and TurboRFP that is transcriptionally controlled by three c-di-GMP-dependent riboswitches; thus TurboRFP expression positively correlates with c-di-GMP abundance [105]. Comparing *P. brasiliense* BF45 and BF20 strains, among which BF45 is the more virulent in the host models of celery and broccoli, authors showed that BF20 produced the highest levels of c-di-GMP and was more motile and less proficient in biofilm formation and exopolysaccharide production [105].

## 4. Visualization of Biocontrol Strategies to Counteract Virulence of *Dickeya* and *Pectobacterium* in Potato Plant and Tuber

### 4.1. Plant Root Colonization and Biocontrol Effectiveness of Fluorescent-Tagged Soft-Rot Pectobacteriaceae Antagonists

Controlling SRP during plant growth, crop storage, and transit has remained a challenge for decades. So far, SRP control is mostly based on the use of certified seed material and the application of hygienic measures during agriculture procedures [26,110]. Until now, there was also a lack of effective strategies to combat *Pectobacterium* and *Dickeya* spp. pathogens, which could be implemented to reduce the economic losses in crop production. The rhizosphere bacteria or plant endophytes may protect plant tissues toward SRP [99,111,112,113,114]. Most of the research on the selection of strains inhibiting the growth of SRP is focused on their mode of action, which includes production of antimicrobials, competition for nutrient and iron ions [115,116,117,118,119,120], and interference with the cell-to cell communication (QS mechanism–see below) of the pathogens [113,121,122,123,124]. Many of these potential BCAs contribute to plant tissue protection via a combination of antagonistic activities, e.g., production of antimicrobials, induction of systemic resistance in plants, and interfering in pathogens’ cell-to-cell communication [108,115]. The majority of research on biological control concerns crop plants (e.g., potato, Chinese cabbage); however, BCAs have also been developed for the control of bacteria in ornamentals [119,124]. Jafra et al. [124] selected *Dickeya zeae* antagonists, which included, among others, the strains of *Rahnella aquatilis* and *Erwinia persicina* from hyacinth bulb tissues. Li et al. [119] studied the effectiveness of *Myxococcus* sp. BS against *P*. *carotovorum* subsp. *carotovorum* on calla lily under greenhouse conditions.

**Figure 2:**
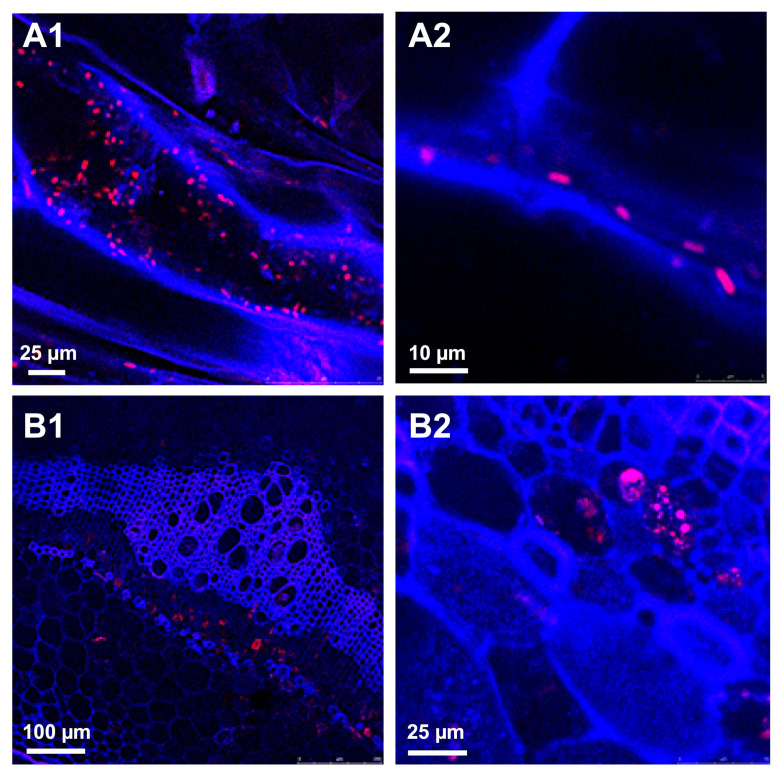
Colonization of potato roots and stems with DsRed-tagged *Dickeya solani.* DsRed fluorescent protein is a relevant alternative to the widely used green fluorescent protein (GFP) for tagging soft-rot *Pectobacteriaceae*. In this example, the peak emission of DsRed (610 nm), very far from that of the autofluorescence of plant cell walls (450 nm), allowed fine localization of *D. solani* IPO3012 (see Table 1) red cells and microcolonies, both in the blue cells and walls of the roots (**A1**,**A2**) and stems (**B1**,**B2**). For technical experimental details please see reference [125]. (Courtesy of Dr. Jan van der Wolf, WUR-PRI, Wageningen, the Netherlands).

Although the number of potential SRP biocontrol strains studied under laboratory condition is growing, there is a limited number of studies evaluating their biocontrol potential under greenhouse, field, or storage conditions [117,118,119,120,125,126], and even less data on the use of FPs in studies of plant colonization efficiency, their use on/in plants, and effectiveness in inhibiting pathogen activity. Notwithstanding, SRPs, as well as the bacterial antagonists of *Pectobacterium* spp. and *Dickeya* spp., have been FP labeled to assess their ability to stably colonize plant tissues and protect plants from infection development. *Serratia plymuthica* strain A30, one of the widely studied potential BCA strains, has been successfully labeled with GFP and used individually or together with DsRed-tagged *D. solani* IPO3012, in a potato mini-tuber co-inoculation study evaluating plant tissue colonization, population dynamics within the plant tissue, and antagonism toward pathogens *in planta* [100]. The effectiveness of the A30 strain against *D. solani* IPO3012 was studied in pot-grown potato plants under greenhouse conditions. The visualization of the plant colonization by both strains applied singly or together was performed by CLSM. This study confirmed that *S. plymuthica* A30 is able to stably colonize potato roots and stems, as the GFP-tagged cells of *S*. *plymuthica* A30 were observed inside of xylem vessels and between parenchyma cells. While applied together, FPs-labelled A30 (GFP-tagged) and *D. solani* IPO3012 (DsRed-tagged), the green (A30) and red (IPO3012) cells were seen seven days after inoculation in roots of the treated plants, both in xylem vessels and between protoxylem cells of the vascular tissue [125]. During the course of the experiment, GFP-tagged cells were still observed both inside roots and stems, but the DsRed cells were not present, yet they were present in the control plants inoculated only with DsRed-tagged *D. solani* (Figure 2). This study confirmed the effectiveness of A30 in the reduction of pathogen populations in potato plants [125]. *S. plymuthica* A30 was also used in the post-harvest treatment of seed potato studies to verify its effectiveness in the protection of seed material during storage and potato plants in the next growing season [127], which proved the efficacy of A30 in the reduction of soft-rot occurrence during tuber storage and blackleg incidence during field cultivation of potatoes.

Another example of the application of GFP-tagged potential BCAs against soft-rot pathogens is a recent study by Cui et al. [120], in which the *Bacillus amyloliquefaciens* KC-1 strain was used to control *P. carotovorum* subsp. *carotovorum* on Chinese cabbage in vitro and *in planta.* GFP-tagged *B. amyloliquifaciens* KC-1 was used to study population dynamics in the Chinese cabbage leaves and rhizosphere under greenhouse conditions. The KC-1 strain was sprayed, and *gfp*-directed qPCR was used to determine the DNA copies of the KC-1 strain per gram of dry soil. This analysis confirmed the ability of *B. amyloliquifaciens* KC-1 to colonize the rhizosphere and leaves of Chinese cabbage and significantly suppressed the growth and reduced the number of *P. carotovorum* subsp. *carotovorum*. The authors underlined the importance of root colonization and establishment of a stable population on the plant roots by potential BCAs [112]. Thus, FPs are important tools for studying the effectiveness of root/stem colonization by potential BCAs. GFP-tagging has been applied to study the ability of three potential BCAs, namely *Bacillus subtilis* MB73/2, *Pseudomonas* sp. P482 (currently *P. donghuensis* P482) and *Ochrobactrum* sp. A44 (currently *O. quorumnocens* A44) to stably colonize the roots of pot-grown potato plants [116,128,129]. In the case of *B. subtilis* M73/2, a *gfp* reporting cassette was introduced into the *amyE* locus in the M73/2 genome via homologous recombination, and the plasmid-borne *gfp* was used to label P482 and A44 strains. The GFP-tagged variants were used to inoculate seed tubers. Four weeks after tuber bacterization, the effect of root colonization was observed using CLSM. *O. quorumnoscens* A44 colonized the potato root more effectively than the remaining two strains. Notably, the A44 strain was originally isolated from the potato rhizosphere [130], yet P482 originated from tomato roots and M73/2 from an uncharacterized weed plant.

As mentioned above, the number of studies referring to the application of FPs in the studies on biological control of SRP is rather limited. The introduction and stable maintenance of the genes encoding FPs, as well as their expression in various environmental bacteria, can be a technical challenge due to broad antibiotic resistance in potential BCAs and difficulties with transformation of the host cells, or inability to stably express FP genes.

### 4.2. Tracking and Interference of Quorum-Sensing Communication Used by Soft-Rot Pectobacteria

Numerous bacteria use cell-to-cell communication systems based on both synthesis and perception of signaling molecules to evaluate population density and synchronize their social behavior [131]. The most-studied system occurs in some Gram-negative bacteria and relies on the production of molecules belonging to the family of AHLs [131]. The production of AHL signals has a significant ecological impact on the rhizosphere. As an example, it has been shown that AHL-communication used by the *Pseudomonas aureofaciens* strain 30–84 and its crosstalk by members of the rhizosphere community may alter its secondary metabolite production and pathogen inhibition involved in the biocontrol of wheat take-all [132,133]. The use of promoter-probe vectors carrying FP reporters has become also a powerful way to localize and quantify AHL-based communication of various bacteria [45,134,135,136]. These biosensors have been constructed to detect a wide range of AHLs, improving the level of sensitivity and/or specificity over the years. Some of them are adapted to the detection of short chain-AHLs, while others are better adapted to long chain or oxo-substituted AHLs. Thus, Steidle and co-workers [46] developed several monitor strains to visualize AHL-mediated communication between bacteria cells throughout colonization of the tomato rhizosphere. Three GFP-based AHL sensors were introduced in plant-associated bacteria, detecting different ranges of AHL molecules (short-medium-long length). Globally, the three systems are based on transcriptional fusion of the *luxI* promoter from the LuxI/LuxR system and a *gfp* gene. They showed that rhizobacteria, which colonize tomato roots, produce AHL signaling molecules that could be perceived by other bacteria. Riedel et al. [136] developed the same work to investigate communication between two AHL producers, *Pseudomonas aeruginosa* and *Burkholderia cepacian,* in biofilms. They showed that *B. cepacia* was able to perceive AHL molecules from *P. aeruginosa*, while the reverse was not observed. As for Steidle’s works, transcriptional fusion was used to monitor AHL molecules, based on the *luxI* promoter positively affected by the product of the *luxR* gene, controlling the expression of the *gfp* reporter. Andersen et al. [45] applied the same strategy with another GFP reporter to study the communication exchange between various bacteria species, namely *P. aeruginosa*, *P. aureofaciens*, *Serratia liquefaciens*, *Serratia ficaria*, and *E. coli*. As another significant advance, the works of Gantner et al. [137] quantified, for the first time, the spatial scale of AHL-mediated cell-to-cell communication between a *Pseudomonas putida* population emitting signals and another one only able to detect them. Image analysis led to the determination of an effective bacterial “calling distance” on tomato root surfaces. This is most frequent at 4–5 μm and can reach up to 78 μm. Finally, Charlesworth et al. [134] developed another fine method, coupling a GFP-based biosensor with thin layer chromatography to increase the specificity and sensitivity of their AHL-biosensor.

**Figure 3:**
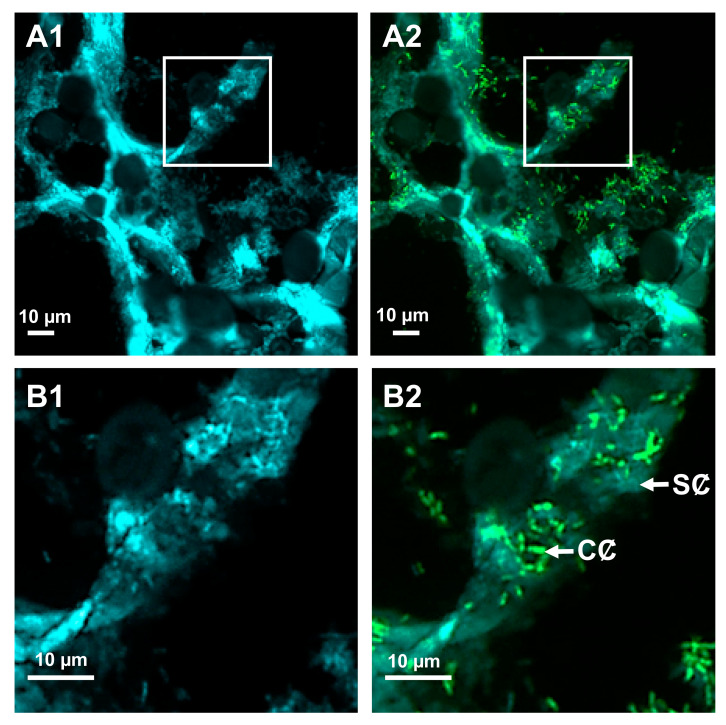
Simultaneous observation of the location and quorum-sensing communication of *Pectobacterium atrosepticum* during potato tuber soft-rot. Tubers were wounded and inoculated axenically with the *P. atrosepticum* CFBP6276 dual-colored strain (see Table 1): the constitutively produced cyan fluorescent protein served as a cell tag (blue fluorescing cells), whereas reporter fusion based on the green fluorescent protein (GFP) enabled the simultaneous recording of the production and presence of *N*-acyl-homoserine lactone (AHL) signaling molecules (green fluorescing cells). Confocal microscopy observations of the bottom surface of the tuber wound were achieved at two days post-inoculation with an ultrathin cross-section. Images were combined in the blue and red channels (**A1**,**B1**), supplemented with the green channel (**A2**,**B2**). In these conditions, slight blue (**A1**,**B1**) or celadon (**A2**,**B2**) autofluorescence revealed the cell wall structure of the pith, as well as translucent parenchyma cells containing opaque and oval amyloplasts. Close-ups (delineated by squares) of clustered and embedded pectobacterial cells in (**A1**,**A2**) are shown in (**B1**,**B2**), respectively. Legend: CȻ, communicating cell, SȻ, silent cell. For technical experimental details please see reference [103].

AHL-based QS controls the expression of more than 1000 genes in *P. atrosepticum*, hence, up to a quarter of its genome, including most of the virulence factors [138]. SRP also used AHL-based QS to coordinate the switch from host primo-invasion to soft-rot steps and trigger the synchronized production of massive amounts of lytic enzymes involved in plant tissue degradation, overwhelming plant defenses [35,37,39,138]. It, therefore, seemed instructive to monitor the AHL production of this bacterium throughout host invasion. Recently, Chane et al. [103,139] assessed the AHL-based communication of *P. atrosepticum* by using a *lux*-based reporter system carried by the pME6000 plasmid. This construct consists of a transcriptional fusion between the *luxI* promoter and *gfp_asv_* gene, encoding the unstable GFP_ASV_ fluorophore, the expression of which is under the control of transcriptional factor LuxR [45]. In the presence of AHLs, the QS sensor LuxR binds to the *luxI* promoter region and activates transcription of the *gfp_asv_* gene (Table 1). The pME6000 plasmid also contains a *cfp* gene, the expression of which is under a constitutive promoter. Therefore, a dual-colored biosensor was then built with the constitutive cyan tag of the bacteria by CFP production and with the inducible *gfp* tag to assess the QS activity using green fluorescence. Thus, by choosing adapted channels, it became possible to simultaneously monitor both the localization of bacteria and its potential QS activity (Figure 3). CLSM analysis of such dual-labeling makes it possible to show the inter- and intracellular colonization of the pith by pectobacteria, and that the latter were preferentially aggregated in the more or less hydrolyzed plant cell wall [103]. Moreover, the switch between primo-colonization and the soft-rot step was accompanied by the production of bright-green fluorescence, reflecting concomitant and strong AHL production. Because the GFP_ASV_ produced by a sensor strain is short lived, green fluorescence reflects the fairly recent AHL induction of *gfp_asv_* gene expression [137], allowing the detection of a cell quorum at two days post-inoculation. This fluorescence occurred in a great part of *Pectobacterium* single cells and microcolonies at the cellular quorum, revealing the coordinated action of the pathogenic population [103,140] (Figure 3).

Since QS is essential for disease progression, disrupting the AHLs exchange between SRP by a quorum-quenching (QQ) mechanism (firstly named quorum-sensing inhibition, QSI) appears to be a promising anti-virulence strategy for plant health [139,141,142,143]. QQ activities are carried out by some microorganisms that are capable of synthesizing acylases, oxidoreductases, and/or lactonases involved in the cleavage of different parts of AHLs [143,144,145,146,147,148]. This is the case in the Gram-positive bacteria *Rhodococcus erythropolis* R138 [149]. This strain is able to effectively degrade a wide range of AHLs and suppress the disease in hydroponic and field culture conditions [149,150]. Full QQ activity of the biocontrol agent R138 requires the expression of the QS degradation (*qsd*) pathway, leading to the production of QsdA and QsdC enzymes, involved in the lactone ring and acyl chain catabolism of AHLs, respectively [121]. Thus, the recent discovery of the regulatory mechanism involved in *qsd* operon expression was a decisive event for the development of the first biosensor for QQ [151]. Indeed, the *qsd* operon is regulated by a TetR-like transcriptional repressor, named QsdR [152,153]. In the absence of the QS signal, this repressor binds to the promoter region of the *qsd* operon, switching off the pathway. When AHL signals are present in the cell environment, their homoserine lactone ring binds to QsdR, thereby changing its conformation. Thus, QsdR cannot bind to the promoter region, inducing derepression of the *qsd* operon expression [151]. To monitor both environmental location and AHLs lysis-based QQ of R138 strain, Barbey et al. [151] constructed a red biosensor tagged with the mCherry fluorophore. The *mcherry* gene was cloned into the pEPR1 vector downstream of the strong constitutive promoter P-45 [154].

To follow the QQ activity, AHL detection and degradation was investigated by introducing a transcriptional fusion between the *qsd* promoter and *gfp*_UV_ reporter gene and the transcriptional factor encoding the *qsdR* gene into the pEPR1-*mCherry* vector. With this construction, *gfp*_UV_ gene expression was under the control of the *qsd* promoter and QsdR regulator, mimicking the chromosomal regulation system of *qsd* operon expression [151]. By using this rhodococcal QQ biosensor and the pectobacterial QS reporter strain described above, Chane et al. [103,140] were able to observe, on the same image, the QS activities of *P. atrosepticum* CFBP6276 with QQ and concomitant tuber protection displayed by the biocontrol agent R138, when the two strains were co-inoculated into the tuber. Here, AHL catabolism was observed inside regions containing a mixture of large amounts of pathogenic (green cells) and biocontrol cells emitting red plus green (i.e., a yellow to amber) fluorescence [103]. The QS and antagonistic QQ activities could also be fully isolated at the resolution limit of CLSM by decomposing the overall image according to the different image channels. This allowed extraction of green fluorescence associated with both the GFP_ASV_ (QS activity) and GFP_UV_ fluorophores (QQ activity) produced by the pectobacterial and rhodococcal cells, respectively, from the blue fluorescing background of the tuber [140]. Besides, the CLSM makes it possible to obtain images in three dimensions. The use of Z-stacks as a component of measuring relationships of cells across different tissue depths is particularly interesting to ensure that there is no overlap and that pathogens and protectors candidates interact on the same plant cell layer. Finally, these biosensors and their well-adapted FPs have unraveled that the QQ activity of the rhodococcal biosensor varied according to cell metabolism and AHL availability and can be quantified. Indeed, the amount of GFP_UV_ fluorophore produced is a function of AHL degradation and the cells exhibit fluorescence that varies from amber to yellow, according to the modulation of GFP_UV_ fluorophore synthesis [140].

## 5. Conclusions

The use of promoter-probe vectors carrying fluorescent protein reporter genes appears to be a relevant way to study the epidemiology and etiology of SRPs (Table 2). This technology, coupled with adequate microscopy, made it possible to verify the bacterial viability in situ and provide direct visual evidence of the colonization kinetics on the host plant, as well as the capacity of SRP to form microcolonies and/or biofilms. The biosensors were also able to reveal, *in planta*, that these pathogens form active clusters embedded in cell wall components that can explain macroscopic damage to the plant. They also unveiled cell-to-cell communication at both the single-cell and microcolony level within the plant host, proving that SRP single cells produce enough AHL signaling molecules to communicate with neighboring cells.

However, these biosensors can suffer from certain limitations. During their exposure in the environment or the plant, they must not lose the plasmids carrying FPs encoding genes. Moreover, they must show sufficient sensitivity and, therefore, sufficient fluorescence to allow their detection, depending on the number of copies of the FP-vector used and the FP selected, in addition to its promoter expression. Finally, color variation in fluorescence can be generated by heterogeneity of FP expression in the sensor population. Such a phenomenon was observed for dual-colored cells, exhibiting a high variability in the expression of the GFP-based reporter in microcolonies [83,103,140]. These variations are related to the fact that the clonal cell populations exhibit substantial phenotypic variation and division-based growth rate diversity [155,156]. Consequently, when a gene encoding an FP was weakly expressed in a dual-tagged strain, the ratio of the two fluorescence varied in the cells of the same population, indicating heterogeneity of the expression of the gene from individual to individual. Typically, this is the case when the expression of one of the reporter genes is directed by a strong constitutive promoter and that of the other one by an inducible promoter [103]. The biosynthesis and intracellular function of FPs are also questionable: for example, their size and tendency to oligomerize can lead to dysfunctional fusion proteins, while oxygen-dependent fluorescence interferes with their use when anaerobic conditions rise [157].

Despite these current technological limitations, the SRP biosensors should also enable the subcellular localization of proteins involved in virulence as was obtained recently in a strain of *Xanthomonas* sp. [158]. The use of the unstable (with short half-lives) variants of the GFP, in this case, allowed precise localization of the viable cells *in planta*, reducing the possibility that the GFP signal is generated from unviable (dead) bacterial cells. The same approach can be used in SRP to determine, in detail, the localization of alive cells in different plant compartments and structures. In conclusion, the use of FPs in the studies targeting the interaction of SRP bacteria with their hosts will have a major impact on the understanding of pathogen interactions with plants on the cell level. Future studies will highly benefit from the already developed, as well as new, FPs and their accompanying visualization tools.

## Figures and Tables

**Table 1:** Fluorescent proteins already used to construct *Dickeya* and *Pectobacterium* tagged strains and biosensors.

Name	Excitation/Emission Used (nm)	Carrier Strain	Plasmid Probe	Application	Reference
GFP	485/505	*D. solani* IPO2254 (bv.3)	pPROBE-AT-*gfp*	Localization, spread	[97,98,99]
GFP	495/505	*D. solani* IPO2253 (bv.1)	pPROBE-AT-*gfp*	Localization, spread	[100]
DsRed	532/610	*D. solani* IPO3012 (bv.3)	pRZ-T3-*dsred*	Localization, spread	[100]
DsRed	557/579	*D. dianthicola* IPO3018 (bv.7)	pRZ-T3-*dsred*	Localization, spread	[100]
GFP	495/505	*D. dianthicola* IPO3019 (bv.7)	pPROBE-AT-*gfp*	Localization, spread	[100]
mCherry	560/615	*P. brasiliense* 1962	pMP7604-*mCherry*	Localization	[101]
Egfp	488/507	*P. brasiliense* 1962	pMP4657-*egfp*	Localization	[29]
Egfp	488/507	*P. brasiliense* 1962ΔexpI ^1^	pMP4657-*egfp*	Localization	[29]
GFP	488/509	*P. brasiliense* SX309	pSMC21-*gfp*	Localization	[102]
CFP and GFP_ASV_	405/477488/509	*P. atrosepticum* CFBP 6276	pME6000-*luxR*-P*_luxI_::gfp_ASV_-cfp*	Localization and endogenous QS detection ^2^	[103]
CFP and GFP_ASV_	405/477488/509	*P. atrosepticum* CFBP 6276-EI ^1^	pME6000-*luxR*-P*_luxI_::gfp_ASV_-cfp*	Localization and exogenous QS detection ^2^	[103]
GFP	495/505	*P. parmentieri* IPO3399	pPROBE-AT-*gfp*	Localization, spread	[104]
AmCyan and TurboRFP	420/450550/580	*P. atrosepticum* SCRI1043	pFY435 ^3^	c-di-GMP production	[105]
AmCyan and TurboRFP	420/450550/580	*P. brasiliense* BF20	pFY435 ^3^	c-di-GMP production	[105]
AmCyan and TurboRFP	420/450 550/580	*P. brasiliense* BF45	pFY435 ^3^	c-di-GMP production	[105]

^1^*luxI* mutant derivative of wild-type strain, unable to produce *N*-acyl homoserine lactone signals; ^2^ QS: *N*-Acyl-homoserine lactone-based quorum-sensing; ^3^ Encodes a c-di-GMP biosensor consisting of two fluorescent reporters: AmCyan with constitutive expression, and TurboRFP that is transcriptionally controlled by three c-di-GMP-dependent riboswitches.

**Table 2:** The first described use of fluorescent proteins (FP) and FP-tagged strains to monitor the fate of SRP bacteria during interaction with plants.

Process	Bacterial Strain	Host Plant	Fluorescent Protein Used	Reference
Bidirectional bacterial movement inside plants	*D. solani* strain IPO2254 *P. carotovorum* subsp. *carotovorum* strains Pcc3 and Pcc13	Potato (*Solanum tuberosum* L.) Sun star (*Ornithogalum dubium*)	GFP GFP	[97,98,106]
Symptomless dissemination of bacteria inside plants	*D. solani* strain IPO2254	Potato (*Solanum tuberosum* L.)	GFP	[97,98]
Colonization of (progeny) tubers	*D. solani* strain IPO2254	Potato (*Solanum tuberosum* L.)	GFP	[97,98]
Presence and spread of bacteria inside tolerant vs. susceptible host	*P. brasiliense* strain *Pcb*1692	Potato (*Solanum tuberosum* L.)	mCherry	[101]
Fate of bacterial strains in salicylic treated host	*D. solani* strain IPO2254	Potato (*Solanum tuberosum* L.)	GFP	[108]
Fate of AHL-deficient bacterial variants in host plant	*P. brasiliense* strain *Pcb*1692	Potato (*Solanum tuberosum* L.)	GFP	[29]
Systemic colonization of alternative plant hosts	*D. solani* strain IPO2254	Bittersweet nightshade (*Solanum dulcamara* L.)	GFP	[107]
Colonization of plants after spray-inoculation	*D. solani* strain IPO2254 *P. parmentieri* strain IPO3399	Potato (*Solanum tuberosum* L.)	GFP	[104]
Detection of quorum-sensing activity	*P. atrosepticum* strain 6276	Potato (*Solanum tuberosum* L.)	GFP and CFP	[103]

## Data Availability

Data is contained within the article and supplementary material.

## Supplementary Materials

The following are available online at https://www.mdpi.com/2076-2607/9/2/295/s1, Table S1: Spectral catalog of fluorescent proteins (FPs) likely to be used to tag bacteria and construct a promoter-probe vector.
