# Supplementary material for: Biosensors Used for Epifluorescence and Confocal Laser Scanning Microscopies to Study Dickeya and Pectobacterium Virulence and Biocontrol
Source: Microorganisms. 2021 Feb 1;9(2):295. doi: 10.3390/microorganisms9020295 (PMC7912877; doi:10.3390/microorganisms9020295)
Supplement: Supplementary file 1 [file microorganisms-09-00295-s001.pdf]

microorganisms

MDPI

Review

Biosensors Used for Epifluorescence and Confocal Laser Scanning Microscopies to Study *Dickeya* and *Pectobacterium* Virulence and Biocontrol

Yvann Bourigault[1,2], Andrea Chane[1], Corinne Barbey[1,2], Sylwia Jafra[3], Robert Czajkowski[4]* and Xavier Latour[1,2,]*

[1] Laboratory of Microbiology Signals and Microenvironment (LMSM EA 4312) - University of Rouen Normandy - 55 rue Saint-Germain, 27000 Evreux, France; yvann.bourigault@univ-rouen.fr (Y.B.); chane.andrea@gmail.com (AC); corinne.barbey@univ-rouen.fr (C.B.)

[2] Research Federations NORVEGE Fed4277 & NORSEVE - Normandy University, F-76821 Mont-Saint-Aignan, France

[3] Division of Biological Plant Protection, Intercollegiate Faculty of Biotechnology UG and MUG, University of Gdansk, ul. A. Abrahama 58, 80-307 Gdansk, Poland; sylwia.jafra@biotech.ug.edu.pl (S.J.)

[4] Division of Biologically Active Compounds, Intercollegiate Faculty of Biotechnology UG and MUG, University of Gdansk, ul. A. Abrahama 58, 80-307 Gdansk, Poland

* Correspondence: robert.czajkowski@biotech.ug.edu.pl ; Tel.: +48 58 523 63 33 (R.C.); xavier.latour@univ-rouen.fr; Tel.: +33-235-146-000 (X.L.)

Supplementary Material

Table S1. Spectral catalog of fluorescent proteins (FPs) likely to be used to tag bacteria and to construct a promoter-probe vector (FPs are classified by ascending order of excitation wavelengths).

Name	Color	Excitation peak (nm)	Emission peak (nm)	Reference
Sirius	Purple	355	424	[40]
Azurite	Blue	383	447	[40]
BFP	Blue	381	445	[50]
EBFP	Blue	380	446	[51]
EBFP2	Blue	383	448	[40]
SBFP1	Blue	380	446	[51]
SBFP2	Blue	380	446	[51]
TagBFP	Blue	402	457	[40]
PS-CFP2	Cyan	400	468	[40]
PS-CFP	Cyan	402	468	[40]
SCFP3A	Cyan	433	474	[52]
SCFP3B	Cyan	433	474	[52]
SCFP2	Cyan	434	474	[52]
mTurquoise	Cyan	434	474	[40]
Cerulean	Cyan	433	475	[40]
ECFP	Cyan	434	477	[40]
SCFP1	Cyan	434	477	[52]

microorganisms

Table S1. *Cont.*

Name	Color	Excitation peak (nm)	Emission peak (nm)	Reference
mECFP	Cyan	434	477	[40]
CyPet	Cyan	435	477	[40]
amajGFP (amFP486)	Cyan	458	486	[53]
mTFP0.7	Cyan	453	488	[54]
CFP	Cyan	458	480	[50]
TagCFP	Cyan	458	480	[40]
AmCyan1	Cyan	458	489	[40]
mTFP1	Cyan	462	492	[40]
MiCy (Midori-Ishi Cyan)	Cyan	472	495	[40]
mUKG	Cyan	483	499	[40]
cFP484	Cyan	456	484	[55]
dstrGFP	Green	456	484	[53]
cgigGFP	Green	399	496	[53]
Anm1GFP1	Green	475	495	[56]
asulGFP (asFP499)	Green	403/480	499	[53]
mUkG1	Green	483	499	[40]
hcriGFP	Green	405	500	[53]
ppluGFP1	Green	480	500	[56]
ppluGFP2	Green	482	502	[56]
TurboGFP	Green	482	502	[40]
pmeaGFP2	Green	487	502	[56]
GFP	Green	395/475	504	[50]
bsDronpa	Green	460	504	[57]
pmeaGFP1	Green	489	504	[56]
Anm1GFP2	Green	490	504	[56]
mAG1	Green	492	505	[40]
Dendra	Green	492	505	[58]
ZsGreen	Green	493	505	[40]
AcGFP1	Green	475	505	[40]
TagGFP	Green	482	505	[40]
TagGFP2	Green	483	506	[40]
zoanGFP (zFP506)	Green	496	506	[53]
scubGFP1	Green	497	506	[53]
scubGFP2	Green	497	506	[53]
laesGFP	Green	491	506	[56]
E0GFP	Green	400	508	[40]

microorganisms

MDPI

Table S1. *Cont.*

Name	Color	Excitation peak (nm)	Emission peak (nm)	Reference
dendGFP	Green	492	508	[53]
ptilGFP	Green	500	508	[53]
EGFP	Green	489	509	[40]
mWasabi	Green	493	509	[40]
EmGFP	Green	487	509	[40]
Emerald	Green	487	509	[59]
Superfolder GFP	Green	485	510	[60]
mEGFP	Green	489	509	[40]
GFP2	Green	≈400	≈510	[40]
rmueGFP	Green	498	510	[53]
T-Sapphire	Green	399	511	[40]
pdae1GFP	Green	491	511	[56]
SGFP1	Green	495	512	[51]
SGFP2	Green	495	512	[51]
Dis3GFP	Green	503	512	[53]
mKikGR	Green	505	515	[61]
IrisGFP	Green	488	516	[58]
GFPa1	Green	497	516	[62]
EosGFP	Green	506	516	[58]
mcavGFP	Green	506	516	[53]
PA-GFP	Green	504	517	[63]
rsFastLime	Green	496	518	[57]
rfloGFP	Green	508	518	[53]
Kaede	Green	508	518	[64]
Dendra2	Green	480	520	[61]
Dronpa	Green	503	522	[57]
Padron	Green	503	522	[57]
TagYFP	Yellow	508	524	[40]
Sapphire	Yellow	400	525	[65]
mAmetrine	Yellow	406	526	[40]
UnaG	Yellow	500	527	[62]
EYFP	Yellow	514	527	[40]
Topaz	Yellow	514	527	[40]
SYFP2	Yellow	515	527	[40]
Venus	Yellow	515	528	[40]

microorganisms

Table S1. *Cont.*

Name	Color	Excitation peak (nm)	Emission peak (nm)	Reference
Cp157Venus	Yellow	515	528	[40]
Citrine	Yellow	516	529	[40]
mCitrine	Yellow	516	529	[40]
YFP	Yellow	≈515	≈530	[40]
Ypet	Yellow	517	530	[40]
phiYFP	Yellow	525	537	[40]
zoanYFP (zFP538)	Yellow	494/528	538	[53]
TurboYFP	Yellow	525	538	[40]
zFP538	Yellow	527	538	[40]
ZsYellow1	Yellow	529	539	[40]
Dendra2	Yellow	440	550	[61]
mBanana	Orange	540	553	[65]
mKO (Kusabira-Orange)	Orange	548	559	[40]
mKO2	Orange	551	565	[40]
mHoneydew	Orange	487	562	[65]
mOrange	Orange	548	562	[40]
mOrange2	Orange	549	565	[40]
TurboRFP	Red	553	574	[40]
rfloRFP	Red	566	574	[53]
dendRFP	Red	557	575	[53]
zoan2RFP	Red	552	576	[53]
mcavRFP	Red	508	580	[53]
IrisRFP	Red	551	580	[58]
Kaede	Red	572	580	[64]
dTomato	Red	554	581	[40]
tdTomato	Red	554	581	[40]
EosRFP	Red	571	581	[58]
DsRed	Red	563	582	[40]
DsRed2	Red	563	582	[40]
TagRFP	Red	555	584	[40]
TagRFP-T	Red	555	584	[40]
DsRed-Express	Red	555	584	[40]
mTangerine	Red	568	585	[65]
DsRed-monomer	Red	556	586	[66]
DsRed-Max	Red	560	589	[40]
DsRed-Express2	Red	554	591	[40]

Table S1. *Cont.*

Name	Color	Excitation peak (nm)	Emission peak (nm)	Reference
mApple	Red	568	592	[67]
AsRed2	Red	576	592	[40]
dis2RFP	Red	573	593	[53]
asCP562	Red	562	595	[62]
PAmCherry1	Red	564	595	[68]
asulCP (asCP)	Red	572	595	[62]
PAmCherry2	Red	570	596	[68]
PAmCherry3	Red	570	596	[68]
mStrawberry	Red	574	596	[40]
Anm2CP	Red	572	597	[56]
KFP1	Red	580	600	[69]
KFP (asFP595)	Red	580	600	[40]
TurboFP602	Red	574	602	[40]
mRuby	Red	558	605	[40]
RFP611	Red	555	606	[40]
mRFP1	Red	584	607	[70]
rsCherry	Red	572	610	[40]
rsCherryRev	Red	572	610	[40]
J-Red	Red	584	610	[56]
KillerRed	Red	585	610	[71]
mCherry	Red	587	610	[40]
eqFP611	Red	559	611	[67]
RFP611	Red	559	611	[67]
asFP595-148G	Red	580	615	[40]
mKeima	Red	440	620	[40]
mRaspberry	far-Red	598	625	[40]
Sandercyanin	far-Red	375	630	[62]
mKate	far-Red	588	635	[72]
mKate2	far-Red	588	633	[40]
Katushka2	far-Red	588	633	[40]
Katushka	far-Red	588	635	[40]
HcRed	far-Red	590	637	[73]
RFP639	far-Red	588	639	[74]
mPlum	far-Red	590	649	[40]
mNeptune	far-Red	600	650	[40]

microorganisms

MDPI

Table S1. *Cont.*

Name	Color	Excitation peak (nm)	Emission peak (nm)	Reference
AQ143	far-Red	595	655	[72]
SmURFP	far-Red	642	670	[62]
